# The clinical significance of open vs. minimally invasive surgical approaches in the management of thymic epithelial tumors and myasthenia gravis

**DOI:** 10.3389/fsurg.2024.1457029

**Published:** 2024-12-11

**Authors:** Nathan J. Alcasid, Ivana Vasic, Phillip G. Brennan, Jeffrey B. Velotta

**Affiliations:** ^1^ San Francisco-East Bay, Department of Surgery, University of California, Oakland, CA, United States; ^2^ Division of Thoracic Surgery, Department of Surgery, Kaiser Permanente Northern California, Oakland, CA, United States; ^3^ Department of Clinical Science, Kaiser Permanente Bernard J. Tyson School of Medicine, Pasadena, CA, United States; ^4^ Department of Surgery, University of California, San Francisco, CA, United States

**Keywords:** thymectomy, thymoma, myasthenia gravis, mediastinum, video-assisted thoracoscopic surgery (VATS), robotic-assisted thoracoscopic surgery (RATS)

## Abstract

Though advancements have been made in the pharmacologic treatment of myasthenia gravis (MG), surgical resection is not only an option as a last line of defense for those patients who do not respond to medical therapy but also remains vital for those with thymic epithelial tumors (TET). While prior studies have shown the potential superiority of minimally invasive approaches via robotic- and video-assisted thoracoscopic surgery (RATS/VATS) for thymectomy compared to open surgery, in the setting of malignancies, this outcome delineation is controversial. As RATS/VATS may be associated with less post-operative complications in the treatment of TET, some surgeons argue that the open approach is necessary for complete resection (R0 resection) and to prevent potential seeding of the malignancy. In this review article, we will compare the efficacy and implications of the different surgical approaches and techniques themselves in performing a thymectomy for autoimmune and oncologic pathologies.

## 1 Introduction

Thymic epithelial tumors (TET) have an incidence of approximately 3 per 1,000,000 patients, and while they are considered relatively rare, they comprise the most common primary tumors of the anterior mediastinum (1–4). TET's are currently classified into three major categories as thymoma, thymic carcinoma, and thymic neuroendocrine tumors (NET) (1). Thymoma is the most common type comprising of 80% of all TETs (1). Though thymic carcinoma and thymic NET are much more rare, they are more aggressive with a 5-year overall survival (OS) of 55% and 28%–78% respectively, compared to 90% with thymomas (5–8).

Thymomas are known to be associated with a myriad of paraneoplastic syndromes such as myasthenia gravis (MG), pure red cell aplasia, hypogammaglobulinemia, and other autoimmune diseases (7). MG is a known autoimmune disease that causes fluctuating weakness in facial, respiratory, and extremity skeletal muscles in affected individuals (9, 10). According to a recent study by Rodrigues et al., the prevalence of MG in the United States is 37 per 100,000 patients with an increasing incidence in younger women (<50 years old) (11). MG has been known to have a distinctive relationship in those with or without the presence of a thymoma. Clinical manifestations are present in approximately 50% of patients with a thymoma and conversely, approximately 10% of patients with clinical signs of MG have an associated thymoma (9, 10).

This distinct relationship between MG and the thymus gland has both clinical and surgical implications. For patients presenting with signs and symptoms of MG in the setting of a confirmed thymoma, surgical resection of the thymus, via a thymectomy, is indicated for definitive treatment (9, 10, 12). However, even for the majority of patients, such as those who present with signs and symptoms of MG but without a thymoma, a well-established therapeutic option remains with performing a thymectomy in the non-diseased thymus gland which allows for improved overall clinical outcomes in addition to reducing long-term pharmaceutical and medical therapy (9, 10, 12).

While thymectomy has been a widely accepted therapy in those with MG and TETs, the optimal surgical approach has remained controversial since its advent. This holds even more particularly true now with evolution of newer minimally invasive techniques. A thymectomy via an open, transcervical approach was first described by Sauerbruch and Roth in 1912 (12). Since then, the transcervical approach has been met with criticisms, particularly in the challenge of obtaining complete negative margins. Proponents of the transcervical approach may argue that the decreased morbidity may be of preference compared to the more invasive transsternal approach and may be reserved for smaller thymic tumors (12, 13). However, when compared to transsternal approach, the transcervical approach avoids an extensive neck and mediastinum dissection required to resect all thymic tissue. Given the lack of complete anatomic exposure and potential for incomplete margins, which remains as the most vital prognostic factor for thymic tumors, the transsternal approach has been the traditional approach (14–16). Since then, significant advancements in the field of surgery have allowed for newer modalities in the thymectomy approach. Minimally invasive approaches are increasingly being utilized ranging from video-assisted thoracoscopic surgery (VATS) and robotic techniques. However, with these emerging VATS and robotic approaches, a plethora of variability within each newer approach still exists from the use of unilateral or bilateral thoracoscopic techniques to the additions of insufflation and energy device usage (13, 17). As the operative treatment for TETs and MG continues to rapidly advance, the purpose of this review is to present and describe the different surgical techniques associated with TET and MG pathologies along with their implications in achieving optimal clinical outcomes with complete R0 resection.

## 2 Thymectomy overview—preoperative assessment

Preoperative assessment for those undergoing thymectomy begins with a routine history and physical with close attention to those who have had prior radiation or surgery to the neck or thorax (4). It is vital to note any historical signs and symptoms associated with other mediastinal tumors such as lymphoma or germ cell tumors (7). For operative planning, a thorough physical exam should be performed to evaluate for any lymphadenopathy as well as limitations of neck extension, chest wall deformities, and/or obesity that would preclude certain thymectomy approaches. In addition to obtaining baseline clinical laboratories, evaluating thyroid function tests, tumor markers, or MG antibodies may be warranted. Preoperative imaging typically includes obtaining a computed tomography (CT) chest with IV contrast to define the anatomical characteristics of the thymic tumor and its relationship to nearby structures. Although there are no consensus guidelines or definitions on determining resectability of a thymic tumor, CT imaging may aid in identifying tumor characteristics and infiltration to nearby intrathoracic structures such as integrity of the thymic capsule, mediastinal fat, pleura, and nearby vascular structures (7, 18). In a study by Hayes et al, the largest predictors of unresectability were degree of abutment of nearby vascular structures and the presence of pleural nodularity (19).

## 3 Thymectomy overview—general guidelines

The Masaoka clinical staging guidelines which had been previously the most commonly used staging system worldwide has now largely been supplanted by the eighth addition of the American Joint Committee on Cancer (AJCC) TNM classification system (1, 4, 5). This newer edition encompasses staging of all thymic malignancies including thymic NETs (20). In general, previous Masaoka stage I and II are consistent with the AJCC guidelines and are still defined as stage I and II and are similarly treated with definitive thymectomy (5, 20). Masaoka stage III tumors are equivalent to AJCC stage IIIa tumors where definitive thymectomy may be pursed if deemed resectable based off imaging or in tumors that require neoadjuvant chemotherapy prior to surgery (7, 20). Thymic tumors that are deemed unresectable are classified by Masaoka stage IVa/b and AJCC stage IIIb/IVa/IVb (5, 20). In a retrospective study evaluating thymomas on CT imaging, Marom et al, found that the largest independent predictors that indicated advanced disease (Stage III/IV) were tumor size >7, mediastinal fat infiltration, and presence of a lobulated contour (21).

## 4 Thymectomy overview—surgical principles

Located in the anterior mediastinum, the thymus gland is encapsulated and has a rich blood supply provided by branches of the internal mammary arteries, inferior thyroid, and pericardiophrenic arteries (1, 7). Anatomically, it has two lobes, each with superior and inferior horns that extend to the bilateral phrenic nerves forming the lateral borders of resection for a total thymectomy. The superior boundary is formed by the thyroid-thymic ligaments and innominate vein and the inferior border is formed by the diaphragm. Total thymectomy to achieve negative margins generally entails resection of the mediastinal pericardium, pleura, ipsilateral pulmonary segments, innominate vein, with or without inclusion of the phrenic nerve. Completion of an R0 resection will allow for optimal oncologic outcomes (3, 22).

For patients with MG, a total thymectomy is indicated and is discussed further below in this review. Surgical management of other TETs have traditionally been managed via a total thymectomy, though partial thymectomy is controversial, its role in smaller and early-stage tumors that are not associated with MG are increasing (14, 23, 24). In a retrospective study evaluating low stage thymomas (stage I and II) undergoing partial vs. total thymectomy, the authors found no difference in disease free and overall survival between the two groups (23). Several additional studies have also demonstrated no oncologic differences in rates of recurrence in patients with low stage tumors undergoing partial vs. total thymectomy (14, 24).

Lymph node dissection is defined by the recent eight edition of the AJCC TNM staging where N1 nodes are defined as the anterior thymic region whereas N2 nodes are defined by the deep thymic region (25). Extent of lymphadenectomy (LAD) is based on thymic tumor infiltration into nearby mediastinal structures. If there is no tumor infiltration, only N1 nodes are required. If there is evidence of infiltration, then sampling of N2 nodes are recommended (25).

### 4.1 Surgical techniques and approaches

**Figure 1:**
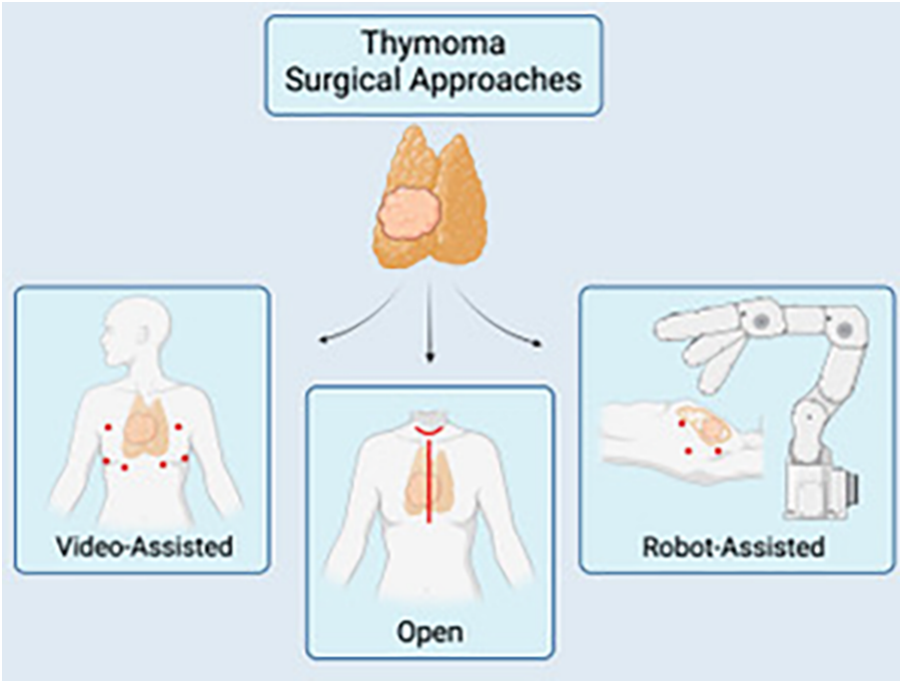
A schematic of the multiple surgical approaches for thymectomy: video-assisted (VATS) (left), open thymectomy via a median sternotomy or transcervical approach (center), and robotic-assisted (RATS). Image created by biorender.com.

Though various approaches and techniques exist to perform a thymectomy, the most optimal approach should be dictated by the ability to achieve the overlying similar goal to resect all thymic tumor tissue while avoiding injury to the recurrent laryngeal, vagus, and phrenic nerves whenever possible. Open surgical options for a thymectomy include the transsternal and transcervical approaches, while newer minimally invasive modalities include the use of VATS or robotic approaches via small incisions (Figure 1). Each approach varies in surgical exposure of overall thymic tissue resection and ability to perform extracapsular dissection. Each exposure will be further discussed here.

#### 4.1.1 Transsternal thymectomy

The transsternal approach is mediated by performing a median sternotomy. It has traditionally been regarded as the standard approach for thymectomies due to its ability to gain wide exposure into both the mediastinum and neck when compared to the open, transcervical approach (12). Compared to the transcervical approach, the transsternal approach is much more suited for removal of large thymic tumors and those that infiltrate nearby mediastinal structures (26, 27).

#### 4.1.2 Transcervical thymectomy

The first reported thymectomy was via the transcervical approach performed via a small transverse or curvilinear neck incision made approximately 2 cm above the sternal notch (12, 28). The transcervical thymectomy allows for a less morbid operation albeit at the expense of a less robust exposure. The inability to fully explore the mediastinum and potential to leave residual thymus tissue during resection has lended the transcervical approach to be less favorable than the traditional transsternal approach. Additionally, this approach requires patients to fully extend their neck. More recently however, this approach has seen promising results when chosen in the treatment for those patients with MG without a thymoma. Multiple retrospective studies have compared the transcervical vs. the transsternal approach in this set of patients demonstrating that the transcervical approach allows for less post-operative morbidity, shorter length of hospital stay, while having similar clinical symptom improvement as those who underwent a median sternotomy (28–30).

#### 4.1.3 Minimally invasive thymectomy

Minimally invasive techniques consist of two different modalities, either via a VATS or use of the robotic approach. Each minimally invasive technique is performed via small port incisions from either the right or left side (or both sides) of the patient (31). Whether the VATS/robotic technique is approached from the left or right side, it is typically standard to utilize three ipsilateral ports with an optional thoracoscopic port on the contralateral side. A contralateral thoracoscopic port is particularly important to maintain view of the contralateral phrenic nerve. The decision to perform a left or ride sided approach is typically dictated by tumor location and characteristics as well as surgeon preference. While a right sided approach may allow for more “operative room” without being obscured by the heart and optimal exposure of the superior vena cava and innominate vein, the left sided approach allows for more mediastinal exposure and dissection. A bilateral approach has also been described as this allows for maximal visualization to remove all necessary mediastinal tissue and preserve bilateral phrenic nerves. The subxiphoid approach was first described in 1999 but has only recently gained more common usage over the past decade, particular in Eastern countries when compared to Western countries (32, 33). As the subxiphoid approach has been refined to a form of a minimally invasive technique, one advantage over other thoracoscopic surgery include ability to maximize view of bilaterally phrenic nerves simultaneously without having to perform a bilateral VATS/robotic surgery. Its use as a single-port approach also has been described to decrease intercostal neuropathy (32, 33).

In addition to fundamental thymectomy resections, VATET (video-assisted thoracoscopic extended thymectomy) has also been described for extended thymectomies with several described approaches for larger tumors; stage III. These include, the single-port subxiphoid approach, the subxiphoid and subcostal arch method, and combination of the subxiphoid and VATS approaches. As expected, the single port approach has the advantage of not entering the chest and thus less risk of pain and intercostal nerve injury (34). However, there has been a highly noted learning curve with requirement of special instruments. Some proponents recommend a combination of subxiphoid, and VATS extended thymectomy without using sternal lifting and utilizing traditional laparoscopic instruments with reduction in post-operative pain and cosmetic wound satisfaction (34).

## 5 Comparison of surgical techniques and approaches

Despite the myriad of open and minimally invasive techniques that exist for thymectomy, whether for MG or for other TETs, there remains no consensus on the optimal approach. While early stage thymomas and thymic carcinomas may guide a more feasible approach to minimally invasive techniques, some surgeons still prefer the open transsternal approaches (35). In this section, we provide the current literature comparing the perioperative and operative outcomes of both open and minimally invasive techniques.

### 5.1 Open vs. minimally invasive approaches

Regardless of the operative approach chosen when dealing with TETs, completion of an R0 resection remains the most vital prognosticator for long term outcomes in addition to local recurrence (14–16, 36). Thus, operating surgeons should base operative technique on the ability to achieve a safe and complete thymectomy. In 2017, Burt et al., performed a review and compared approximately 2,500 patients over 15 years who underwent thymectomy either via the open or minimally invasive approach (37). After propensity-matching for each operative technique group, they found no statistical difference in the ability to perform an R0 resection (37). They also found no statistical differences in perioperative mortality between the two groups (37). Additionally, on multivariable regression, the operative technique was not found to be an independent predictor on the ability to achieve an R0 resection (37). Their study found that lower tumor stage and absence of radiotherapy as independent predictors in performing a complete resection (37). One criticism, however, was the inability to determine and compare effects on locoregional recurrence as the patients who underwent minimally invasive techniques were not followed on a long-term basis. Thus, while short-term results are promising for minimally invasive thymectomy in performing a complete resection, more longitudinal studies are required to determine the efficacy in minimizing long term locoregional occurrence.

While congruency between open and minimally invasive techniques remain in comparison of oncologic outcomes, when evaluating additional perioperative outcomes, minimally invasive techniques have demonstrated progress in decreasing perioperative morbidity. Several studies, including meta-analyses, have demonstrated a statistically significant decrease in length of hospital stay with one study demonstrating almost a 50% decrease (1.9 days compared to 4.6 days) with use of VATS thymectomy over the transsternal approach (10, 28). Kang et al. compared the use of robotic thymectomy to the transsternal approach and found that only 1% suffered post-operative complications compared to the 12% who underwent the transsternal approach. This finding was mainly due to the fact that sternal transection was mitigated, and thus the rates of mediastinitis were lower in the robotic approach (38). Respiratory function and pulmonary complications were also found to be more persevered in those who underwent VATS compared to the transsternal technique. Ruckert et al., found quicker recovery of forced vital capacity (FVC) and forced expiratory volume/sec (FEV) by post-operative day three in those undergoing minimally invasive techniques over the transsternal approach (39). Another feared operative complication is the rate of nerve injury which may have devastating post-operative sequela. Unfortunately, there is not enough current literature to confer which approach may be the most optimal in minimizing this complication and is chiefly based on anecdotal evidence and surgeon experience.

Minimally invasive techniques have also shown promise with mitigating myasthenic symptoms in those with MG. There have been multiple studies demonstrating the long-term efficacy of the VATS and robotic approaches in providing relief of MG symptoms with low recurrence rates (10, 40, 41). Yano et al., performed a prospective study on those undergoing thymectomy for early stage thymomas and found no statistically significant difference in post-operative myasthenic crisis between VATS and the transsternal approach, where incidence was approximately 4% for both (42).

### 5.2 VATS vs. robotic approaches

As VATS and robotic approaches are starting to gain notoriety in the treatment of MG and TETs, current data is premature to determine and substantiate the superior minimally invasive approach (43, 44). In one of the most recent comprehensive studies over 20 years comparing VATS and robotic techniques in the treatment of thymomas, authors from the Mayo Clinic found that VATS was associated with a statistically significant reduction in blood loss (65 ml vs. 160 ml) and quicker operative times (102 min vs. 178 min) compared to the robotic approach (44). There were no differences found in length of stay or perioperative mortality (44). The authors found that perioperative complications (presence of phrenic nerve palsy, pericarditis, atrial fibrillation, and pleural effusion) were lower for the robotic approach at 9% compared to 16% for the VATS approach, though these findings were not statistically significant (44). In a systematic review and meta-analysis performed by O'Sullivan et. al, which compared robotic thymectomy to both open and VATS techniques, they found the robotic approach to be superior to the transsternal approach in obtaining a lower positive margin rate while comparable to VATS in post-operative complication rates, length of stay, and operative times (13). Longer periods of evaluation post-operatively though are needed to determine their efficacy in affecting oncologic outcomes. Another important consideration when comparing these approaches is also the rate of conversion to open. Current data suggests that the incidence of conversion rates range from 0%–7%, though this data is mainly based on case series (17, 45, 46). Ruckert et al. performed a retrospective review comparing VATS and robotic approaches to thymectomy and found no significant differences in conversion to open rates with VATS at 1.3% and robotic at 1.4% (39). Proponents of the robotic technique believe that this newer approach, with increased visualization and dexterity, allow it to have more potential than its VATS counterpart and potentially to be standard technique for early stage thymomas (17). Like VATS, surgeons can approach dissection from either side. Some experts report that one way to optimize the learning curve of performing robotic surgery is to perform it via a right-sided approach due a larger operative field including visualization of the venous confluence and aortocaval groove without cardiac interference (17). Though choice is typically guided by the overall anatomy of the thymus tumor where left sided approaches allow for better visualizations of the left innominate vein and aortopulmonary window which aid in identifying sites of ectopic thymus (17). The right sided approach may also not be ideal when performing left innominate vein dissection when identifying nearby thymic veins which may be found along the upper left horn of the thymoma (17). Some experts have identified possible radiological criteria to guide preference for minimally invasive approaches: lesions smaller than 5 cm, anterior mediastinal location with encapsulation, presence of adequate fat plane between thymoma and other structures without mass effect, and presence one-sided thymoma extension (17).

## 6 Conclusion and future considerations

Despite the advancements made in treating those with TETs and MG, the optimal surgical approach remains allusive. In addition to the need for more robust prospective and longitudinal studies, the goal of universal guidelines is contingent on how clinicians define and classify the staging of TETs which has changed over recent years (4, 20). Bergh et al. first described the clinical staging for thymomas in 1978 with subsequent modifications by Masaoka in 1981 (5, 47). Previously, use of the Masaoka clinical staging guidelines were accepted and dictated the optimal treatment for thymomas (1, 4). However, since then, the International Association for the Study of Lung Cancer and the International Thymic Malignancy Interest Group have developed a newer staging system in the eight edition of the tumor, node, metastasis (TNM) classification by the AJCC in attempts to ameliorate prior ill-defined treatment algorithms to be in conjugation with newer minimally invasive techniques from the previous Masaoka staging systems (1). This is important as growing literature demonstrates that minimally invasive surgery not only has improvement over open surgery regarding myasthenia gravis-associated symptoms but as well improving quality of life with shorter hospital stay and decreased pain (1, 4). Nevertheless, use of consistent guidelines to classify TETs may be the fundamental step in dictating the best type of surgery we can offer to our patients particularly as our surgical advancements continue to evolve.
